# Diroximel Fumarate-Loaded Solid Lipid Nanoparticles (DRF-SLNs) as Potential Carriers for the Treatment of Multiple Sclerosis: Preformulation Study

**DOI:** 10.3390/ijms262411827

**Published:** 2025-12-07

**Authors:** Debora Santonocito, Giuliana Greco, Maria Grazia Sarpietro, Aurélie Schoubben, Claudia Sciacca, Giuseppe Romeo, Katia Mangano, Carmelo Puglia

**Affiliations:** 1 Department of Drug and Health Sciences, University of Catania, Viale Andrea Doria 6, 95125 Catania, Italy; giuliana.greco@phd.unict.it (G.G.); gromeo@unict.it (G.R.); capuglia@unict.it (C.P.); 2 NANOMED—Research Centre for Nanomedicine and Pharmaceutical Nanotechnology, Department of Drug and Health Sciences, University of Catania, 95125 Catania, Italy; 3 Department of Pharmaceutical Sciences, University of Perugia, Via del Giochetto 5, 06122 Perugia, Italy; aurelie.schoubben@unipg.it; 4 Department of Chemical Sciences, University of Catania, Viale Andrea Doria 6, 95125 Catania, Italy; claudia.sciacca@unict.it; 5 Department of Biomedical and Biotechnological Sciences, University of Catania, Via Santa Sofia 97, 95123 Catania, Italy; katia.mangano@unict.it

**Keywords:** diroximel fumarate, multiple sclerosis, lipid nanoparticles, nose-to-brain administration

## Abstract

Diroximel fumarate (DRF) is an orally administered prodrug used in multiple sclerosis (MS) treatment. Although it exhibits better gastrointestinal (GI) tolerability than its analogues, many patients still discontinue therapy due to frequent GI adverse events. To overcome these limitations, alternative drug delivery systems that bypass the GI tract are needed. Direct nose-to-brain delivery represents a promising approach to circumvent the blood–brain barrier and target the central nervous system; however, limited nasal mucosal absorption and the small volume of the nasal cavity pose significant challenges. Solid lipid nanoparticles (SLNs) can potentially overcome these obstacles by enhancing drug bioavailability and protecting against enzymatic degradation. This research aimed to develop an innovative intranasal nanoformulation of DRF to improve brain targeting and patient compliance. DRF-loaded SLNs were prepared using a solvent-diffusion technique with stearic acid as the lipid phase and Poloxamer 188 as the surfactant. The obtained nanoparticles displayed favorable technological characteristics, with a mean diameter of 210 nm, a polydispersity index of 0.17, and a zeta potential of −36 mV, suggesting good long-term stability. Interactions between SLNs and biomembrane models (MLV) were also studied to elucidate their cellular uptake mechanism. Future work will focus on evaluating the in vivo efficacy of this novel nanoformulation.

## 1. Introduction

Multiple sclerosis (MS) is a chronic autoimmune and inflammatory disease affecting the central nervous system (CNS), where the immune system attacks the myelinated axons, leading to progressive neurological damage [1,2]. MS progression varies, with patients often experiencing episodes of neurological deficits that can become persistent over time [3,4].

Nowadays, there is no definitive therapy for MS; many treatments, particularly for the relapsing-remitting (RR) form, induce significant remission but require chronic administration, inevitably leading to loss of efficacy and/or toxicity [5,6].

**Figure 1:**
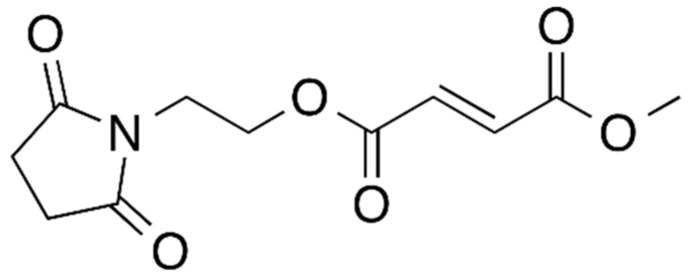
Chemical structure of diroximel fumarate (DRF).

Fumarates have recently attracted a remarkable scientific interest for the treatment of MS. Dimethyl fumarate (DMF) and diroximel fumarate (DRF) are both prodrugs administered orally in MS patients [7]. DMF is cleft by plasma esterases and transformed into the pharmacologically active metabolite, called monomethyl fumarate (MMF), and methanol (MeOH) [8]. Although characterized by a favorable benefit–risk profile, 50% of the patients discontinue the treatment due to gastrointestinal (GI) adverse events (i.e., diarrhea, nausea, vomit and abdominal pain). This has led to the development of the DRF (Figure 1), a new treatment option for relapsing multiple sclerosis (approved by the Food and Drug Administration in 2019) and relapsing-remitting multiple sclerosis (approved by the European Medicines Agency), both as a first and second-line therapy, with a better tolerability profile compared to DMF [9].

However, to be considered a significant treatment choice, further studies need to clarify whether patients with initial or ongoing tolerability issues with DMF will benefit from switching to DRF. The mechanism of action of DRF in multiple sclerosis, mediated by MMF obtained from ester hydrolysis [10], is associated with its anti-inflammatory action through the attenuation of pro-inflammatory cytokine and chemokine production [11,12]. In vivo, a decrease in Th1/17 cells, CD4/8 memory T cells, plasmablasts, IgD memory cells, CD138 plasma cells, and CD56 NK cells were observed, along with an increase in anti-inflammatory Th2 cells, naive CD4/CD8 cells, naive regulatory T cells, transitional B cells, and CD56 bright NK cells [13]. Despite these effects, the low release of MeOH (<10%) is considered the determining factor in the onset of GI side effects, although they are reduced compared to those of DMF [14]. Therefore, it is necessary to formulate a suitable drug delivery system able to bypass the GI tract and allow a controlled and targeted drug release to the brain [15]. Direct nose-to-brain delivery has emerged as a promising strategy for targeting the brain, bypassing the blood–brain barrier and minimizing systemic side effects. However, several challenges remain with nasal drug delivery, including insufficient drug absorption due to the limited surface area of the nasal mucosa and the small volume of the nasal cavity [16]. Although numerous formulations have been developed to address these limitations, drug delivery through the nasal route is still hindered by these anatomical and physiological barriers. Nanocarriers, such as liposomes, lipid-based nanoparticles, and polymeric nanoparticles, have been explored to improve drug permeability and reduce therapeutic doses and side effects, thus enhancing patient compliance [17,18,19]. These nanosystems offer several advantages, including controlled and targeted drug release, which are crucial for brain-targeted therapies [20,21]. Several nanocarrier systems have already been explored for the delivery of fumarate derivatives, particularly DMF, using hybrid nanoparticles or lipid-based formulations [22,23,24]. These studies show that nanosystems can improve tolerability and, in some cases, support CNS targeting [25]. However, their results cannot be directly transferred to DRF, which differs from DMF in structure, stability, and metabolism, and therefore requires a specific formulation approach. Solid lipid nanoparticles (SLNs) have shown promise as effective intranasal carriers for a range of neuroactive drugs, thanks to their biocompatibility and potential to support direct transport to the brain. SLNs are composed of natural physiological lipids, such as triglycerides and fatty acids, which contribute to their excellent stability, ease of production, and lower costs compared to other nanoparticle systems. SLNs offer several advantages, including improved physical stability, reduced toxicological risks, and controlled drug release, which can enhance drug bioavailability by extending drug action, reducing enzymatic degradation, and increasing solubility and permeation [26,27,28].

Moreover, SLNs are generally considered safe, as they are made from biodegradable and physiologically recognized materials.

Despite these promising characteristics, no studies have yet reported the encapsulation of DRF in SLNs or investigated their potential for nose-to-brain delivery. This gap in the literature is significant, considering the unique properties of DRF, which require tailored delivery approaches. Our study aims to address this gap by conducting the first preformulation evaluation of DRF-loaded SLNs for intranasal administration.

The goal of this preliminary work is to develop a suitable SLN formulation for DRF that can improve brain targeting and increase patient compliance in the treatment of multiple sclerosis (MS). Initially, DRF was synthesized and purified in the lab to reduce the high cost of the pure standard. Then, DRF-SLN formulations were prepared and characterized for their mean size, zeta potential, size distribution, and encapsulation efficiency. Furthermore, the ability of SLNs to act as effective carriers for DRF and their mechanism of entry into cells were evaluated through Differential Scanning Calorimetry (DSC) analysis, studying the interaction between SLNs and biomembrane models (MLVs).

## 2. Results and Discussion

### 2.1. Preparation of Diroximel Fumarate

**Scheme 1:**
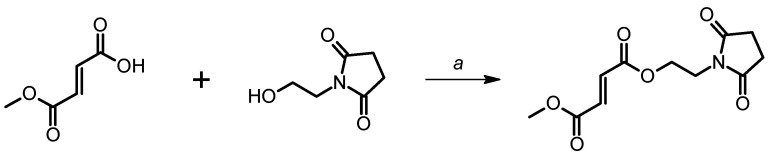
(a) EDAC∙HCl, DMAP, dichloromethane, 30 min, <10 °C; t. a., 20 h.

Since the pure standard (DRF) is very expensive, the preliminary goal was to synthesize this compound in the laboratory in order to minimize costs. Preparation of diroximel fumarate, 2-(2,5-dioxopyrrolidin-1-yl)ethyl methyl (2E)-but-2-enedioate, was accomplished following a literature procedure with some minor modifications [29]. Briefly, (2E)-4-methoxy-4-oxobut-2-enoic acid was reacted with 1-(2-hydroxyethyl)pyrrolidin-2,5-dione in the presence of EDAC-HCl and DMAP to obtain the desired diroximel fumarate (Scheme 1). After recrystallization from ethyl acetate, the pure product was characterized by ^1^H-NMR and ESI/MS; the signals obtained were consistent with the structure of DRF. In TLC analyses, using three mobile phases of different polarity, it proved to be identical to a diroximel fumarate reference standard (MedChemExpress, HY-100375/CS-0018703, Lot # 42553) (see Supplementary Materials).

### 2.2. Characterization of SLNs Loaded with Diroximel (DRF-SLNs)

DRF-SLNs were prepared by solvent-diffusion method using stearic acid as lipid matrix. The choice of this material was previously determined by an in vitro assay on neuronal cell line (primary adult stem cell human dental pulp, DPSCs) [30]. In addition, stearic acid has been shown to be the most suitable lipid for encapsulation of DMF, an analogue of DRF [22,31]. The high encapsulation efficiency observed for the DRF-SLN formulation (74.3 ± 2.9%) supported this choice. In addition, the use of Lutrol^®^ F68 as surfactant is important to obtain small nanoparticles [22]. As reported in Table 1, PCS data showed good technological parameters in terms of mean diameter with a range from 120 to 210 nm, polydispersity index (PDI) values around 0.17–0.26, and zeta potential (ZP) values with a range from −24 to −36 mV. The difference in terms of mean particle size (~100 nm) between blank SLNs and DRF-SLNs was probably due to the DRF encapsulation into nanoparticles [22]. Furthermore, PDI values of both nanoformulations were between 0.17 and 0.26, indicating that the production method used was effective to obtain uniform particles in terms of size distribution. The mean particle size of the DRF-loaded SLNs (~200 nm) falls within the range generally considered favorable for nasal uptake through the olfactory pathway, while the negative ZP values contribute to colloidal stability by reducing aggregation during storage and upon administration. Therefore, these features suggest that the formulation is stable and well suited for nasal delivery, with properties that may facilitate interaction with the mucosal surface and efficient transport to the CNS. In this research, for the first time, we encapsulated DRF in an intranasal nanoformulation to minimize the problems related to free DRF and intranasal administration via conventional formulation [14,16].

**Figure 2:**
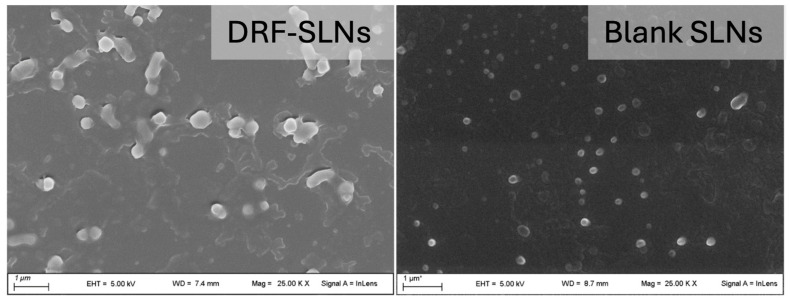
SEM image of DRF-SLNs and blank SLNs.

SEM photomicrograph of DRF-SLNs reveal particles with a mix of rod-shaped and spherical particles and smooth surface (Figure 2). Rod-shaped particles may be ascribed to some particle aggregation during sample preparation for SEM analysis. Blank SLN (Figure 2) photomicrograph shows spherical particles that appear smaller than DRF-SLNs. In addition, the nanoformulation exhibits hydrodynamic sizes that are consistent with those measured by Photon Correlation Spectroscopy (Zetasizer Nano-ZS90).

### 2.3. Differential Scanning Calorimetry (DSC) Analysis

#### 2.3.1. SLNs and DRF-SLNs Calorimetric Analysis

**Figure 3:**
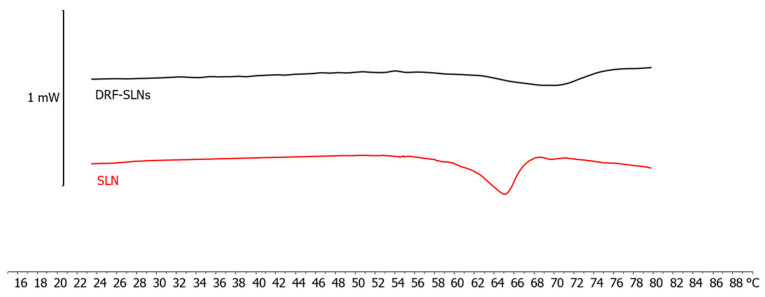
Calorimetric curves, in heating mode, of blank SLNs and DRF-SLNs.

DSC is a thermal analysis technique used to investigate the thermal properties, phase transitions, and changes in enthalpy of various materials, including phospholipid bilayers, which act as nanocarriers and model lipid membranes. This method measures and compares the heat flow of a sample and a reference pan subjected to heating and cooling scans at the same temperature and atmospheric pressure [32]. The difference in heat flow between the sample and reference, recorded as a peak, can be attributed to various processes occurring in the sample, such as phase transitions. The resulting thermograms provide valuable thermodynamic parameters, which can be identified in the calorimetric curves, including the transition temperature (T_m_), which is the point where the heat capacity (C_p_) reaches its maximum value; and the calorimetric enthalpy change ΔH, determined by integrating the area under the peak. DSC has been extensively used to study thermodynamic properties and parameters change and to obtain information about the interaction and reactions. Therefore, blank SLNs and DRF-SLNs were subjected to calorimetric studies to characterize their thermotropic behavior. The differences in the DSC thermograms of blank versus loaded nanoparticles were used to elucidate the interaction between the drug and the lipid components of the nanoparticles. This analysis enabled the assessment of whether the drug had been effectively incorporated into the nanoparticle matrix. Blank SLNs and DRF-SLNs calorimetric curves are shown in Figure 3.

The calorimetric curve associated with blank SLNs exhibited a peak at approximately 65.20 °C, which occurs at a lower temperature than the melting point of the bulk lipid (stearic acid, 69.3 °C) [33]. This observation is likely attributed to the nanocrystalline size of the lipids, thereby confirming the formation of SLN crystallization tendency and polymorphic transitions in triglyceride nanoparticles. The enthalpy variation associated with the SLN curve is −6.00 J/g. The addition of the DRF during the preparation of the SLNs resulted in a notable variation of the thermogram compared to that of the blank nanoparticles. Specifically, the variation in enthalpy was reduced, changing from −6.00 J/g to −5.02 J/g, and the peak became broader and flatter and shifted to higher temperatures, reaching 70 °C. As reported in the literature [34], a decrease in the absolute enthalpy values, combined with peak broadening and flattening, is indicative of a lower crystallinity of the lipid matrix and reduced cooperativity. This reduction in crystallinity reflects the presence of structural defects within the SLN, generated upon drug incorporation [35]. Moreover, the upward shift of the main transition peak indicates that DRF interacts with the hydrophobic core of the SLNs, stabilizing the structure. As a result, the temperature of the main phase transition increases [36]. The behavior observed in our analysis indicates a potential interaction between the active compound and the SLN and suggests that the DRF was effectively incorporated into the lipid core of the nanoparticles.

#### 2.3.2. Kinetics Experiments: Interaction Between MLV and DRF-SLNs

**Figure 4:**
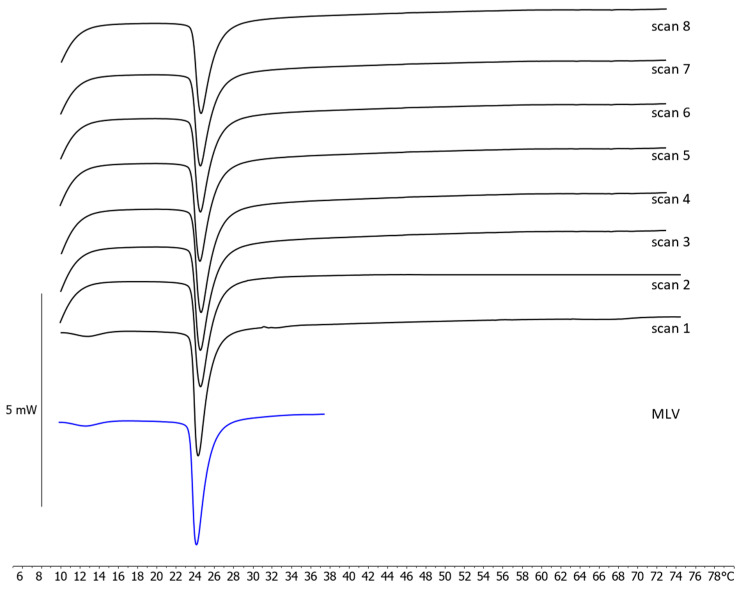
Calorimetric curves, in heating mode, of DRF-SLNs and MLVs placed in contact at increasing incubation time (scan 1–scan 8). The MLV curve refers to the MLV analyzed before they were put in contact with the SLN.

DSC is used to investigate the interaction between the nanocarrier and a biomembrane model, specifically MLVs, which were DRF-SLNs and DMPC MLVs, respectively. MLVs provide a simple model to study early interactions between DRF-SLNs and lipid membranes, but they cannot capture the full complexity of cellular membranes, such as lipid asymmetry or protein-mediated functions. Despite these limitations, they still offer useful insights into the initial nanoparticle–membrane interactions relevant for intranasal delivery. DMPC bilayers are widely used as a biomembrane model due to their T_m_ of approximately 24 °C, which is lower than the physiological temperature (37 °C) at which the experiments are conducted [37,38]. At this temperature, the bilayers are in a disordered liquid crystalline state, facilitating interactions with the drug [39]. The calorimetric curve of the DMPC MLVs is characterized by pre-transition peaks at about 12 °C, related to the transition from the gel state (ordered) to the ripple phase. Furthermore, the main peak at 24 °C was observed, due to the transition from the ripple phase to the liquid-crystalline state, as shown in Figure 4.

When DMPC MLVs were brought into contact with DRF-SLNs and analyzed by DSC, during the first scan (scan 1), both the pretransition and the main transition peaks of DMPC were retained. However, from the second scan onward (scan 2–scan 8), the pretransition peak disappeared, and the enthalpy associated with the main peak decreased, passing from −31.6 J/g to −27.1 J/g (Figure 4).

**Figure 5:**
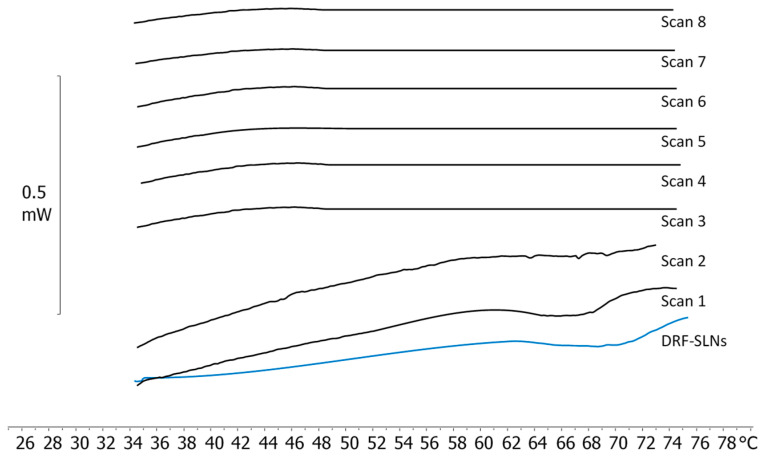
Expanded frame of Figure 4 from 34 to 75 °C. The DRF-SLNs curve refers to the DRF-SLNs analyzed before they were put in contact with the MLV.

Regarding the peak associated with DRF-SLNs, as illustrated in Figure 5 (scan 2), it retains its initial shape while shifting to slightly lower temperatures before eventually disappearing completely, as the contact time increases.

The pretransition peak observed in the MLV thermogram (Figure 4) is associated with the reorientation of the phospholipid head groups and the surrounding water molecules [40]. Therefore, its disappearance suggests interactions between the SLNs and the bilayer, leading to a rearrangement of the phospholipids. The reduction in the enthalpy of the main transition peak is associated with an increase in disorder within the phospholipid membrane caused by external interactions that disrupt lipid packing [41], which can also be attributed to the interaction with DRF. Finally, the flattening of the DRF-SLN peak after contact with MLV indicates that the lipid matrix of the SLN has lost its crystallinity [42], becoming a disordered system. As reported in previous studies, the variation of the MLV peaks combined with the unchanged SLN peak was interpreted as evidence of interaction while preserving nanoparticle structure [43]. In our study, the disappearance of the SLN peak suggests a deeper interaction, likely the fusion with the MLV, rather than simple insertion. Overall, the variations observed in both thermograms confirmed the interaction between the two systems. The results obtained for the interactions between DRF-SLNs and the bio-membrane model indicate that SLNs may be able to penetrate the biomembrane, thus facilitating DRF permeation into the biomembrane itself.

### 2.4. In Vitro Release Study

**Figure 6:**
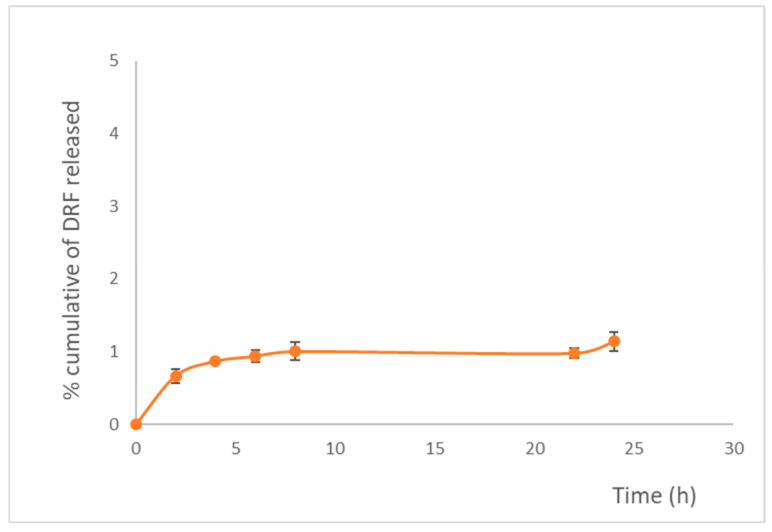
In vitro release profiles of DRF from SLNs Each point represents the mean. value ± S.D. (n = 2).

DRF release from SLNs showed a markedly sustained and limited profile over 24 h. Cumulative release remained below 2% throughout the experiment, with a slight increase during the first few hours (reaching approximately 1% by 8 h), followed by a prolonged plateau up to 22 h. Only a minimal further increase was observed at 24 h (1.14 ± 0.13%), suggesting a highly controlled and retarded release mechanism (Figure 6). The limited release likely reflects the strong affinity of DRF for the lipid matrix and may result from transport of intact SLNs along the olfactory and trigeminal pathways, as suggested by in vivo studies reporting enhanced brain delivery of drugs encapsulated in SLNs and other lipid nanocarriers after intranasal administration [44,45]. The absence of a significant burst effect further supports efficient drug entrapment within the SLN core rather than surface adsorption. These findings are consistent with the low solubility of DRF in aqueous media and highlight the importance of formulation strategies to modulate drug release. Since most literature evidence is based on drug biodistribution rather than direct nanoparticle visualization, these in vitro results require confirmation through further in vivo studies.

## 3. Materials and Methods

### 3.1. Materials

Lecinol S-10, hydrogenated lecithin, was gifted from Nikko Chemical (Milan, Italy) and Lutrol^®^ F68 (MW 8400 g/mol) was obtained by BASF Chem-Trade GmbH (Burgbernheim, Germany). (2E)-4-methoxy-4-oxobut-2-enoic acid, 4-dimethylaminopyridine (DMAP), 1-(2-hydroxyethyl)pyrrolidin-2,5-dione, 1-ethyl-3-(3ʹ-dimethylaminopropyl)carbodiimide hydrochloride (EDAC∙HCl), sodium bicarbonate (NaHCO_3_), hydrochloric acid (HCl), sodium sulfate anhydrous (Na_2_SO_4_), acetone, stearic acid, hydroxypropyl methylcellulose (HPMC K15M), 1,2-dymiristoyl-phosphatidyl-choline (DMPC) and all reagents were purchased from Merck (Milan, Italy). 2-(2,5-dioxopyrrolidin-1-yl)ethyl methyl (2E)-but-2-enedioate (diroximel fumarate) was obtained by D.B.A. Italy s.r.l. (Milan, Italy).

### 3.2. Preparation of SLNs Loaded with Diroximel (DRF-SLNs)

DRF-loaded SLNs (DRF-SLNs) were formulated by the solvent diffusion method, using stearic acid as the lipid phase and Lutrol^®^ F68 (Poloxamer 188) as the surfactant. The choice of using stearic acid as the lipid matrix was based on previous in vitro assays performed on the neuronal cell line Dental Pulp Stem Cell (DPSC) that demonstrated its safety [30]. Firstly, the aqueous phase was prepared by solubilizing hydroxypropylmethylcellulose (0.05 g), soy lecithin (0.05 g) and Lutrol^®^ F68 (0.05 g) in hot water (70 °C). Then, the molten lipid phase, composed of DRF (20 mg/mL), stearic acid (0.005 g) and ethanol, was dispersed in the hot aqueous phase under stirring (1400 rpm) for 8 min. Subsequently, the obtained emulsion was ultrasonicated using an ultrasonic processor (UP 400 S, Dr. Hielscher GmbH, Stuttgart, Germany) for 10 min and cooled in an ice bath for 5 min. Finally, the nanoformulation was filtered and vacuum-treated to ensure uniform dispersion and remove residual ethanol. Blank SLNs were formulated with the same experimental procedure without the addition of DRF.

### 3.3. Characterization of SLNs Loaded with Diroximel (DRF-SLNs)

The mean particle size (Z-Ave) and polydispersity index (PDI) were determined by Photon Correlation Spectroscopy (PCS) using a Zeta Sizer Nano-ZS90 (Malvern Instrument Ltd., Worcs, UK). Before analysis, both nanoformulations were previously diluted with distilled water (1:9 *v*/*v*). The instrument was equipped with a solid-state laser having a nominal power of 4.5 mW with a maximum output of 5 mW 670 nm. Measurements were performed in triplicate at 20 ± 0.2 °C. The zeta potential (ZP, ξ) was measured by Electrophoretic Light Scattering (ELS) using the same instrument and experimental procedures. The morphology of DRF-SLNs was examined using scanning electron microscopy (SEM) with a Field Emission SEM (LEO 1525 equipped with a GEMINI column, ZEISS, Oberkochen, Germany). For SEM analysis, samples were prepared by placing a drop of the diluted nanoparticle suspension onto a circular microscope slide, which was then mounted onto an aluminum specimen stub with a double-sided adhesive carbon disk. The samples were sputter-coated with chromium prior to imaging (100 mA, 24 s, 8 nm thickness) using a Quorum Q150T ES sputter coater (East Grinstead, West Sussex, UK). The encapsulation efficiency (EE%) of DRF-SLN was determined using a methodology based on Sephadex-LH20 fractionation [46]. Precisely, DRF-SLNs (0.2 mL) was pipetted onto Sephadex-LH 20 column (1.0 × 8 cm) eluting with 4 different solvent mixtures, thus affording 4 fractions: water (15 mL, F1), water:ethanol 50:50 (15 mL, F2), acetonitrile (15 mL, F3), and acetone (10 mL, F4). The collected fractions were dried and re-dissolved in acetonitrile:acetone 50:50 and injected into an HPLC-UV system (Agilent 1100 series, Waldbronn, Germany) connected to a Luna C-18 (Phenomenex, Aschaffenburg, Germany; 5 µM, 250 mm × 4.60 mm) column and eluted with a water:acetonitrile 65:35 mobile phase at a flow rate of 1 mL/min for 20 min. The DRF was quantified at 220 nm and a pure sample of DRF (98%) was employed to build the calibration curve with concentration ranging from 0.1 to 0.7 mg/mL (R^2^ = 0.9998). Aqueous fraction, F1 contained the amount of DRF loaded into SLNs. The EE% was obtained using the following equation (1):

EE% = (mgDRF_1_ ÷ mgDRF_tot_) × 100 (1)

where DRF_1_ is the amount of drug quantified by HPLC-UV injection from the aqueous fraction and DRF_tot_ is the total amount of drug employed for the formulation preparation. The EE% was obtained from the analysis of three independent samples following the same procedure, and the results were statistically analyzed and reported as means ± SD.

### 3.4. Multilamellar Vesicle (MLV) Preparation

Multilamellar vesicle (MLV) liposomes were employed as a biomembrane model to evaluate the interactions with both loaded and blank nanoparticles. MLV were prepared using the thin film hydration technique, as described by Russo et al. [47], as follows:

(1) 50.4 mg of 1,2-dymiristoyl-phosphatidyl-choline (DMPC) were dissolved in 1 mL of chloroform:methanol (1:1, *v*/*v*);
(2) the solvents were evaporated under a nitrogen stream to obtain a thin film on the wall of the vial;
(3) the lipid film was obtained under reduced pressure for 2 h to remove any residual solvents;
(4) 600 μL of 50 mM Tris buffer solution (pH 7.4) were added to the film, followed by heating for one minute at approximately 37 °C, which is about 10 °C above the gel-liquid crystalline phase transition temperature of DMPC (24 °C). The mixture was then vortexed for one minute;
(5) the sample was heated for one minute at 37 °C (which is about 10 °C above the gel-liquid crystalline phase transition temperature of DMPC (24 °C)) and vortexed for one minute;
(6) point (5) was repeated three times;
(7) finally, the resulting milky white suspension was maintained at 37 °C for one hour.

### 3.5. Differential Scanning Calorimetry (DSC)

DSC studies were carried out with a Mettler TA STAR^e^ System equipped with a DSC-1 calorimetric cell and the Mettler TA-STAR^e^ software (16.00 version). DSC analysis was performed in high-sensitivity mode. The DSC was calibrated, in temperature and enthalpy changes, using indium (≥99.95% purity grade), stearic acid (≥99.5% purity grade), and cyclohexane (≥99.99%), based on the instrument set up (DSC 1 Mettler TA STAR^e^).

#### 3.5.1. MLVs, Unloaded and DRF-SLNs Calorimetric Analysis

Unloaded and DRF-SLNs were subjected to calorimetric analysis with the aim of studying their thermotropic behavior. 120 μL of each sample, blank and DRF-SLNs, was put into the DSC aluminum pan and submitted to the analysis under nitrogen flow (70 mL/min), following this procedure: a heating scan from 5 to 85 °C at a rate of 2 °C/min, and then a cooling scan from 85 to 5 °C at a rate of 4 °C/min. Each scan was performed at least three times to check the reproducibility of the results. Also, MLVs were characterized with DSC analysis as follows: they underwent a heating scan from 5 to 37 °C at a rate of 2 °C/min and a cooling scan from 37 to 5 °C at a rate of 4 °C/min; each scan was repeated at least three times to confirm the reproducibility of the data.

##### Kinetics Experiments: Interaction Between MLVs and DRF-SLNs

To obtain information on the mechanism of the eventual interaction, DRF-SLNs (60 μL) were weighed in the calorimetric pan, and an equivalent volume of MLV sample (totaling 120 μL) was added. Then, the pan was hermetically sealed, and the interactions were monitored through DSC analysis under a continuous flow of nitrogen (70 mL/min) as follows: a heating scan from 5 to 80 °C at 2 °C/min, followed by a cooling scan from 80 to 37 °C at 4 °C/min. This was followed by an isothermal period of one hour at 37 °C, and finally, a cooling scan from 37 to 5 °C at 4 °C/min. Each experiment was repeated three times.

### 3.6. In Vitro Release Study

DRF release from SLNs was assessed using Franz diffusion cells (LGA, Berkeley, CA, USA) equipped with cellulose acetate membranes (0.2 µm pore size, 25 mm diameter; Sartorius, Göttingen, Germany). The receptor compartment (4.5 mL) was filled with a hy-droalcoholic solution (H_2_O/EtOH, 80:20 *v*/*v*) and maintained at 37 °C under continuous magnetic stirring. A 400 µL aliquot of the formulation was applied to the donor compartment, ensuring uniform and direct contact with the membrane surface. At predetermined time points (0, 2, 4, 6, 8, 22, and 24 h), 200 µL samples were withdrawn from the receptor phase and replaced with an equal volume of fresh medium to maintain sink conditions. The samples were dried and solubilized in acetonitrile (0.1 mL) and DRF concentration in the collected samples was quantified by HPLC-UV analysis at 220 nm [48], using the same chromatographic method described in the previous Section 3.4.

### 3.7. Data Analysis

All findings are expressed as mean ± standard deviation (SD), based on experiments performed in triplicate (n = 3). Statistical differences between blank and DRF-loaded SLNs were evaluated using Tukey’s test, with significance defined as *p* < 0.05. Data analysis was performed using OriginPro 2024 software (Northampton, MA, USA).

## 4. Conclusions

Diroximel fumarate (DRF), an oral prodrug for Multiple Sclerosis (MS), often causes gastrointestinal adverse events that lead to therapy discontinuation. To address this, we formulated DRF-loaded Solid Lipid Nanoparticles (DRF-SLNs) for nose-to-brain delivery, aiming to improve brain targeting and patient compliance. DRF was synthesized and purified, with 1H-NMR confirming its identity. The DRF-SLNs, prepared via solvent diffusion, exhibited favorable characteristics: a mean diameter of 210 nm, PDI of 0.17, and ζ-potential of −36 mV, indicating good long-term stability. DSC analysis further demonstrated effective interaction between the SLNs and biomembrane models. Based on these promising results, the next objective will be to assess the pharmacokinetic advantages of this nanoformulation, particularly its ability to enhance brain targeting and reduce gastrointestinal side effects, with the aim of improving patient adherence to MS treatment.

## Figures and Tables

**Table 1:** Physicochemical properties of unloaded and DRF-SLNs measured by Photon Correlation Spectroscopy (PCS).

	Blank SLNs	DRF-SLNs
Mean Particle Size (nm)	123.2 ± 0.929 *	210.3 ± 2.458 *
PDI	0.179 ± 0.004	0.179 ± 0.033
Zeta Potential (mV)	−24.3 ± 1.453 **	−36.4 ± 2.431 **

Every value was calculated as the mean (±SD) of three sample measurements (n = 3). * *p* < 0.001; ** *p* < 0.01.

## Data Availability

Data is available on the request from the corresponding authors.

## Supplementary Materials

The following supporting information can be downloaded at: https://www.mdpi.com/article/10.3390/ijms262411827/s1.
