# Supplementary material for: Diroximel Fumarate-Loaded Solid Lipid Nanoparticles (DRF-SLNs) as Potential Carriers for the Treatment of Multiple Sclerosis: Preformulation Study
Source: Int J Mol Sci. 2025 Dec 7;26(24):11827. doi: 10.3390/ijms262411827 (PMC12732671; doi:10.3390/ijms262411827)
Supplement: Supplementary file 1 [file ijms-26-11827-s001.zip › ijms-3961071-supplementary.pdf]

Diroximel fumarate loaded Solid Lipid Nanoparticles (DRF-SLNs) as Potential Carriers for the Treatment of Multiple Sclerosis: Preformulation Study

Debora Santonocito [1,2,*], Giuliana Greco [1,2,*], Maria Grazia Sarpietro [1,2], Aurelie Schoubben [3], Claudia Sciacca [4], Giuseppe Romeo [1,2], Katia Mangano [5], Carmelo Puglia [1,2]

[1] Department of Drug and Health Sciences, University of Catania, Viale Andrea Doria 6, 95125 Catania, Italy; debora.santonocito@unict.it (D.S.); mg.sarpietro@unict.it (M.G.S.); giuliana.greco@phd.unict.it (G.G.); gromeo@unict.it (G.R.); capuglia@unict.it (C.P.)

[2] NANOMED—Research Centre for Nanomedicine and Pharmaceutical Nanotechnology, Department of Drug and Health Sciences, University of Catania, 95125 Catania, Italy;

[3] Department of Pharmaceutical Sciences, University of Perugia, Italy, Via Del Liceo 1, 06123, Perugia, Italy; aurelie.schoubben@unipg.it (A.S.)

[4] Department of Chemical Sciences, University of Catania, Viale Andrea Doria 6, 95125 Catania, Italy; claudia.sciacca@unict.it (C.S.)

[5] Department of Biomedical and Biotechnological Sciences, University of Catania, Via Santa Sofia 97, I-95123 Catania, Italy; katia.mangano@unict.it (K.M.)

Supplementary material

Synthesis of 2-(2,5-dioxopyrrolidin-1-yl)ethyl methyl (2E)-but-2-enedioate (diroximel fumarate)

(2E)-4-methoxy-4-oxobut-2-enoic acid (1.00 g, 7.69 mmol), DMAP (0.05 g, 0.41 mmol), 1-(2-hydroxyethyl)pyrrolidin-2,5-dione (1.42 g, 9.96 mmol) and 20 mL anhydrous dichloromethane were placed, under stirring, in a 50 mL flask in an ice bath (< 10 °C). After 10 minutes, EDAC·HCl (1.53 g, 8.00 mmol) was added and the suspended solids dissolved. The reaction mixture was left at a temperature < 10 °C for 15 minutes and then at room temperature for 20 h.

Subsequently, the reaction mixture was placed in a separating funnel and washed in succession with H_2O (2 x 25 mL), 5% $NaHCO_3$ (2 x 25 mL), 1 M HCl (1 x 25 mL), brine (2 x 25 mL). The organic phase was dried over anhydrous Na_2SO_4 for 12 h. By evaporation of the solvent at reduced pressure, an oil was obtained which solidified over time into a clear amorphous mass (1.23 g).

The crude solid obtained was solubilized at room temperature in acetone (25 mL) to which activated carbon was added. The mixture was stirred for 30 minutes and subsequently filtered; volatiles were eliminated under reduced pressure to obtain a white powder (1.05 g).

The solid obtained was suspended in H_2O (30 mL); the mixture was kept under stirring for 3 h and then filtered to obtain a white powder which was dried under reduced pressure (0.74 g, 38%).

The white solid was finally recrystallized from ethyl acetate to obtain (0.303 g, 16 %) of pure 2-(2,5-dioxopyrrolidin-1-yl)ethyl methyl (2E)-but-2-enedioate (diroximel fumarate), as white crystalline solid.

The product thus obtained was successfully compared by TLC with a diroximel fumarate reference standard (MedChemExpress, HY-100375/CS-0018703, Lot # 42553).

Analyses were performed on TLC aluminium sheets coated with silica gel 60 and a fluorescent indicator (Merck, Silica gel 60 F_{254}), using three mobile phases of different polarity (ethyl acetate/cyclohexane 5/5 v/v; ethyl acetate/cyclohexane 7/3 v/v; ethyl acetate); spots were visualized with a UV lamp (Spectroline, model ENF-240C/FE) at 254 nm. In chromatographic runs with each of the three mobile phases, the product obtained showed a unique spot with identical retention factor (R_f) to the reference standard (R_f = 0.27, ethyl acetate/cyclohexane 5/5 v/v; R_f = 0.47, ethyl acetate/cyclohexane 7/3 v/v; R_f = 0.66, ethyl acetate).

^{1}H-NMR analysis of the synthesized compound was conducted in a deuterated chloroform ($CDCl_3$) solution using a Varian Inova Unity 500 spectrometer. Signals (s, singlet; d, doublet; t, triplet; m, multiplet) of the ^{1}H-NMR spectrum (Figure S1) were consistent with the structure.

^{1}H-NMR (500 MHz, $CDCl_3$): δ 6.83 (d, *J* = 16.0 Hz, 1 H, CH=CH), 6.79 (d, *J* = 16.0 Hz, 1 H, CH= CH), 4.36 (t, *J* = 5.5 Hz, 2 H, OCH_2CH_2N), 3.84 (t, *J* = 5.5 Hz, 2 H, OCH_2CH_2N), 3.80 (s, 3 H, CH_3O), 2.74 - 2.70 (m, 4 H, pyrrolidin-2,5-dione).

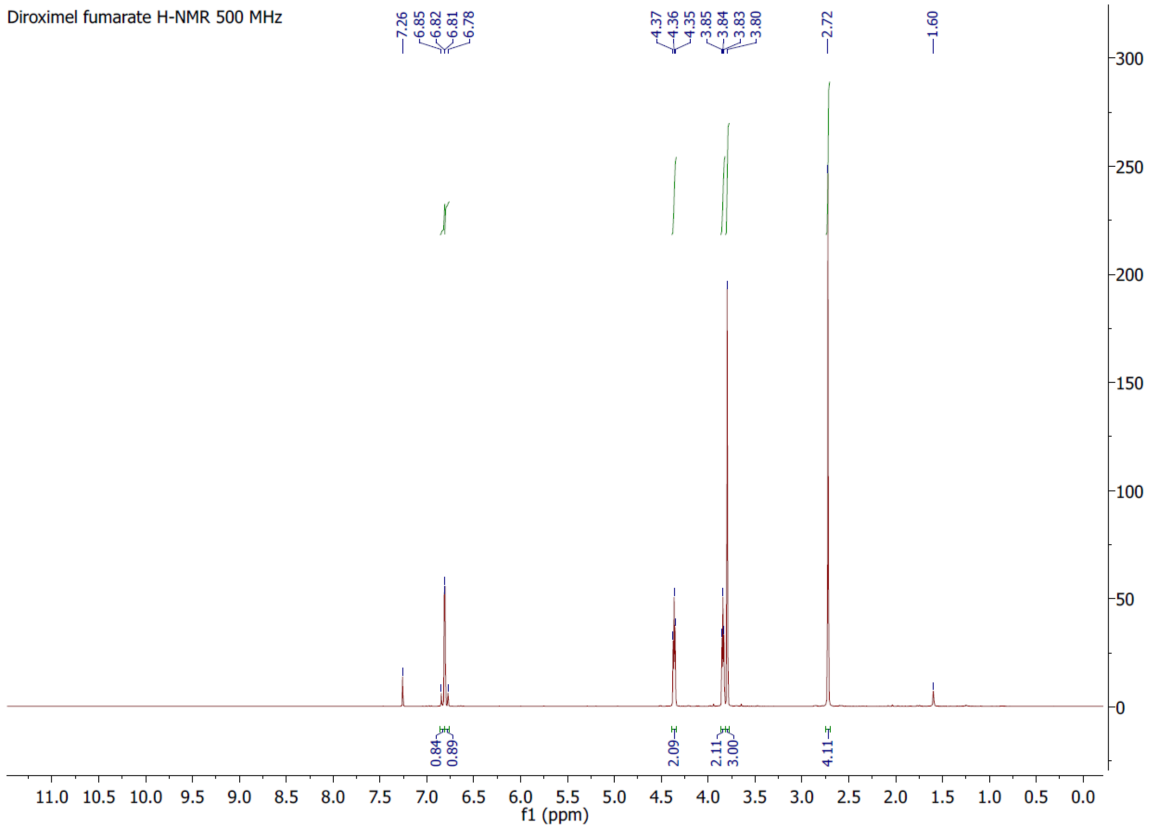

Figure S1. ^{1}H-NMR spectrum of obtained Diroximel fumarate

Mass spectrometry spectrum (Figure S2) of synthesized diroximel fumarate was obtained using the LTQ XL mass spectrometer, equipped with H-ESI II source, (ThermoFisher, San Jose, CA, USA) in full scan mode from m/z 150–500 and in positive mode with a spray voltage of 2–3 kV. The capillary temperature was set to 250 °C, the capillary voltage to 20 V and the tube lens to 120 V. External calibration was performed using the Pierce LTQ ESI Positive Ion Calibration Solution. Data proceeding was performed using the FreeStyle Software ver. 1.6 SP1 (Thermo Fisher Scientific).
ESI/MS m/z: [$M+H^+$]: 256.27, [$M+NH_4^+$]: 273.26, [$M+Na^+$]: 278.35, [$M+MeOH+H^+$]: 287.71, [$M+K^+$]: 295.90; calcd. for $C_{11}H_{14}NO_6^+$ [$M+H^+$] m/z: 256.23.

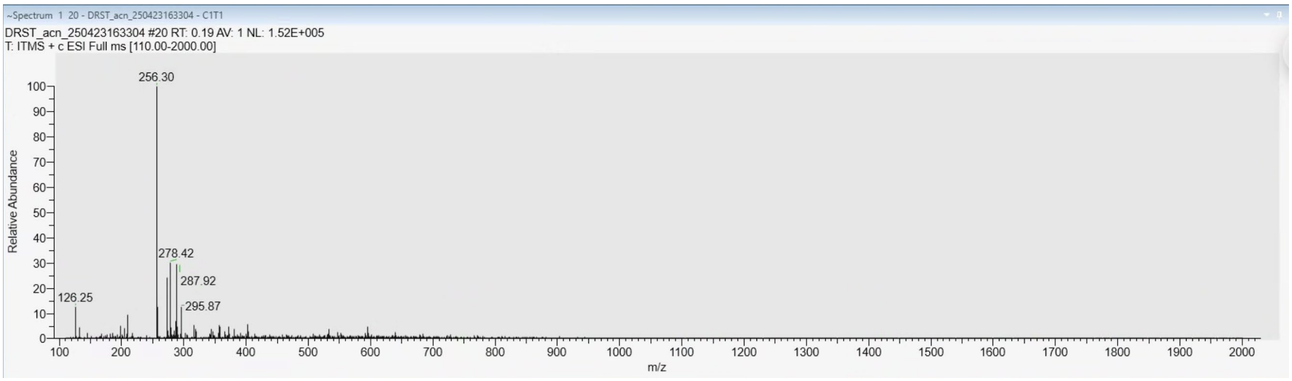

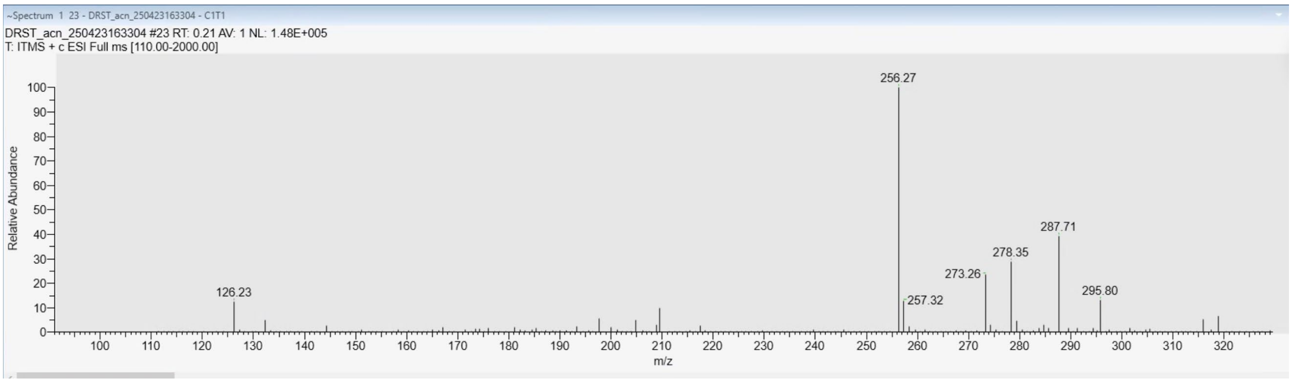

Figure S2. ESI/MS spectrum of obtained Diroximel fumarate (upper panel). X-axis expansion of ESI/MS spectrum (lower panel).
